# Algorithms in *Allergy*: Diagnosis of Concomitant Inducible Laryngeal Obstruction in Adults With Asthma

**DOI:** 10.1111/all.70425

**Published:** 2026-06-19

**Authors:** Andrew P. Hearn, Claire Slinger, Jennifer M. Butler, Jemma Haines, James H. Hull, Stephen J. Fowler

**Affiliations:** ^1^ Department of Respiratory Medicine Barts Health NHS Trust London UK; ^2^ Lancashire Teaching Hospitals NHS Foundation Trust, Preston, Lancashire and Clinical Audiology, Speech and Language Research Centre Queen Margaret University Edinburgh UK; ^3^ Department of Respiratory Medicine Northumbria NHS Foundation Trust Newcastle upon Tyne UK; ^4^ NIHR Manchester Biomedical Research Centre Manchester University NHS Foundation Trust Manchester UK; ^5^ Respiratory Medicine, Royal Brompton Hospital Institute of Sport, Exercise and Health (ISEH) London UK; ^6^ Division of Immunology, Immunity to Infection and Respiratory Medicine, School of Biological Sciences The University of Manchester Manchester UK

Asthma affects around 7% of the adult population and is characterised by episodic cough, wheeze and breathlessness resulting from variable airflow obstruction [1]. Type 2 (T2) inflammatory pathways underlie the most prevalent and best characterised asthma endotypes, and inform diagnostic and therapeutic paradigms. Around 10% of patients have difficult‐to‐treat asthma, in which persistent symptoms or exacerbations occur despite guideline‐directed management, prompting escalation of inhaled corticosteroids (ICS), repeated courses of systemic corticosteroids, and initiation of biologic therapy [1].

Inducible laryngeal obstruction (ILO) is an important and under‐recognised contributor to poor symptom control in asthma. ILO refers to inappropriate, transient and reversible laryngeal narrowing at the glottic or supraglottic level, predominantly during inspiration, resulting in disrupted airflow and acute respiratory symptoms [2]. The clinical significance of expiratory vocal fold narrowing, which is also often seen on laryngoscopy, is not yet fully understood but can occur as a physiological response to lower airway obstruction and is not, in isolation, diagnostic of ILO. The pathophysiology of ILO is incompletely understood but likely initiated by inappropriate laryngeal sensory responses and amplified by cortical influences such as cognitive, emotional and psychological causes. Commonly shared comorbidities include gastro‐oesophageal reflux and post‐nasal drip [3, 4].

Both ILO and asthma manifest with symptoms of breathlessness, cough and wheeze. Consequently, ILO is frequently misdiagnosed as difficult asthma, leading to inappropriate treatment escalation and prolonged morbidity. In asthma, the prevalence of concomitant ILO has been estimated at approximately 30% [5], while asthma represents the most common comorbidity identified in patients enrolled in a UK national ILO registry, with a reported prevalence of 68% [4]. Therefore, clinicians should consider ILO early in the assessment of difficult asthma.

The distinct pathophysiological mechanisms and anatomical sites involved in asthma and ILO give rise to important differences in symptom presentation that are critical for diagnostic differentiation. In asthma, airway hyperresponsiveness, mucosal oedema and mucus plugging occur in the small to medium airways. Consequently, symptoms are predominantly expiratory in nature and typically evolve over hours to days [1]. ILO symptoms are usually inspiratory in nature and may be accompanied by dysphonia, throat tightness, or abnormal throat awareness, reflecting the laryngeal origin of airflow limitation [5]. They typically have an abrupt onset over seconds to minutes and often (but not always) resolve rapidly and spontaneously, consistent with a neuronal mediated pathophysiology. Unlike in asthma [1], ILO does not respond to corticosteroids [5]. Clinical features that can distinguish symptoms of asthma and ILO are shown in the Table 1.

**TABLE 1:** Clinical features supporting the differentiation of asthma and inducible laryngeal obstruction.

Parameter	Features favouring asthma	Features favouring ILO	Clinical utility/limitations
*Symptoms and history*			
Symptom onset	Gradual (hours—days)	Sudden (seconds—minutes)	One of the most useful historical discriminators
Symptom resolution	Gradual (days)	Rapid (minutes—hours)	NB, in some cases ILO can set off laryngeal symptoms that can persist for days
Triggers	URTI, allergens, cold/dry air, exercise	Aerosols, temperature change, exercise, talking, laughing, smoke, emotional stress, strong smells	Although triggers can overlap, the symptoms that they induce will differ
Upper airway symptoms	Absence strongly favours asthma but can be present in absence of ILO	Throat tightness, abnormal throat sensation, voice change, inspiratory noise	Strongly suggestive of ILO. In a single‐centre cohort, dysphonia was associated with a more than two‐fold increased odds of laryngoscopy‐confirmed ILO [6].
Nocturnal symptoms	Symptoms show diurnal variation with nocturnal wakening	Not diurnal, related more to exposures	Sudden nighttime laryngeal symptoms require consideration of laryngopharyngeal reflux, obstructive sleep apnoea and night terrors (seen in post‐traumatic stress disorder)
*Lung function and biomarkers*			
Spirometry	Expiratory airflow obstruction on spirometry/FVL	Flattened inspiratory limb	Normal spirometry does not exclude either condition
Bronchodilator reversibility	Present		Supports asthma but absence does not exclude it
Bronchial challenge	Positive	Negative—may be positive if laryngeal irritation triggered (especially with mannitol)	Useful to request full FVL during challenge if ILO suspected to rule out false positive tests, where progressive flattening of the inspiratory limb will be noted
Type 2 biomarkers	Raised eosinophils and/or FeNO	T2‐low	Absence of T2 biomarkers, especially during exacerbations, should raise suspicion for other causes, including ILO
Intubation: ventilatory pressures	High airway pressures	Low/normal airway pressures	Unexpectedly low (or normal) airway pressures following endotracheal intubation support ILO as a possible diagnosis in patients escalated to mechanical ventilation
*Asthma medication response*			
Systemic corticosteroids	Clear resolution of symptoms: typical time course 24–72 h	No improvement or atypical response (i.e., within minutes or after the course has finished)	Strong discriminator
Inhaled corticosteroids (ICS)	Improvement with optimised therapy	Little or no symptom improvement	Poor response despite adherence should prompt reassessment
Symptomatic response to rapid acting bronchodilators	Symptomatic relief	Symptomatic relief in some cases.	Poor discriminator. Although sometime inhalers (especially dry powders) can provoke ILO
Response to biologic therapy	Reduced exacerbations and improved symptom control	Persistent symptoms/exacerbations	Lack of response should raise suspicion of alternative or co‐existing diagnoses
*Laryngoscopy*			
Laryngoscopy during symptoms	Normal—vocal cord adduction on expiration can be seen in patients with significant lower airways obstruction.	Paradoxical vocal fold adduction on inspiration	Diagnostic gold standard for ILO [2]

*Note:* Summarises clinical, physiological, biomarker, and treatment‐response features that may assist in distinguishing asthma from inducible laryngeal obstruction (ILO). Features should be interpreted as supportive rather than diagnostic and considered within the broader clinical context. Definitive diagnosis of ILO requires laryngoscopic visualisation of inappropriate laryngeal narrowing during symptomatic periods or provocation [2].

Abbreviations: FeNO, fractional exhaled nitric oxide; FVL, flow–volume loop; ICS, inhaled corticosteroids; ILO, inducible laryngeal obstruction; T2, type 2; URTI, upper respiratory tract infection.

Figure 1 presents a pragmatic diagnostic algorithm for identifying co‐occurring ILO in asthma. In such cases, asthma must be confirmed or refuted objectively with evidence of variable expiratory airflow obstruction [7, 8]. ILO may occur in both T2‐high and T2‐low asthma. Uncontrolled T2 inflammation is associated with morbidity; therefore, assessment of ongoing T2 inflammatory activity is a critical early step in the evaluation of symptomatic patients. Other treatable traits, including airflow obstruction, persistent airways infection, smoking or vaping, should be recognised and addressed. Where evidence of uncontrolled T2 inflammation is present, optimisation of inhaled corticosteroid delivery and/or initiation of biologic therapy should be undertaken prior to, or in parallel with, investigation for ILO. Currently, validated screening tools for ILO do not exist and represent an important research need.

**FIGURE 1:**
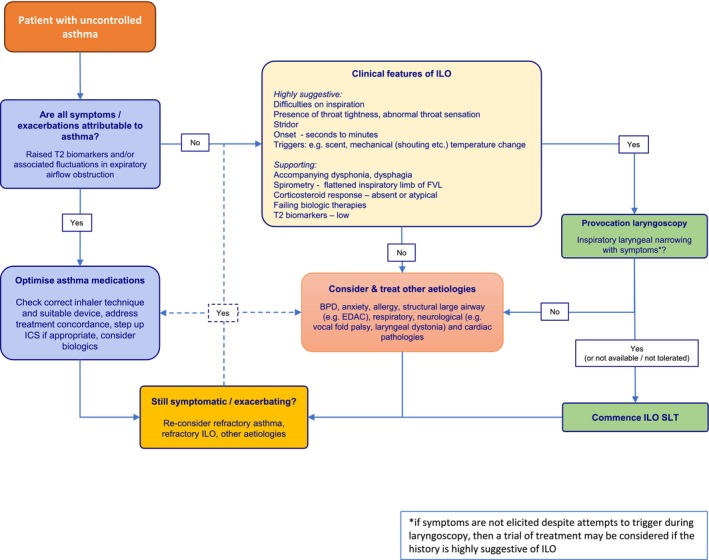
Diagnostic algorithm for identifying co‐occurring inducible laryngeal obstruction (ILO) in patients with asthma. The algorithm applies to patients with a confirmed diagnosis of asthma who remain symptomatic or experience recurrent exacerbations despite guideline‐directed management. In the symptomatic patient, T2 inflammation levels should be assessed and asthma therapy options optimised. Following optimisation of asthma therapy, the presence of clinical features suggestive of ILO—see Table 1—should prompt targeted investigation. Spirometry and flow–volume loops provide supportive but non‐diagnostic information. Definitive diagnosis requires laryngoscopy performed during symptoms or with provocation, demonstrating inappropriate inspiratory laryngeal narrowing. Confirmation of ILO should lead to referral for specialist speech and language therapy‐based interventions, alongside assessment and treatment of breathing pattern disorder. Where provocation laryngoscopy is not available or cannot be tolerated, a pragmatic trial of therapy can be initiated. Alternative causes of breathlessness should be kept in mind throughout patient evaluation. BPD, breathing pattern disorder; EDAC, excessive dynamic airway collapse; Eos, eosinophils; FVL, flow–volume loop; ICS, inhaled corticosteroids; ILO, inducible laryngeal obstruction; SLT, speech and language therapy; T2, type 2.

Physiological investigations have a supportive but limited role. Spirometry is frequently normal in ILO. A flattened inspiratory limb of the flow–volume loop suggests extra‐thoracic obstruction and is present in around 35% of ILO cases [4]. Bronchial challenge testing should be positive in asthma but may provoke ILO in affected patients [9, 10].

Definitive diagnosis of ILO requires laryngoscopy performed during symptomatic periods or with provocation, demonstrating inappropriate transient inspiratory laryngeal narrowing [2]. Laryngoscopy is also important to assess for laryngopharyngeal reflux, gastro‐oesophageal reflux, rhinitis and post‐nasal drip, and to exclude other structural issues such as vocal fold palsy or structural airway occlusion. Excessive dynamic airways collapse and tracheobronchomalacia may also be observed on laryngoscopy although these ILO‐mimics are best evaluated by bronchoscopy.

When ILO is confirmed, management should prioritise non‐pharmacological interventions. Contributory conditions including anxiety, laryngopharyngeal reflux, gastro‐oesophageal reflux, rhinitis/post‐nasal drip, and breathing pattern disorder should be assessed and optimised. Specialist speech and language therapy (SLT)‐led laryngeal control therapy is the mainstay of treatment and should be offered alongside continued optimisation of asthma therapy where indicated. In patients with refractory symptoms, multidisciplinary review should be considered, including consideration of a psychological assessment. If provocation laryngoscopy is not available, a pragmatic and monitored trial of SLT can be employed. Further detail on the components of provocation SLT‐led intervention is provided in the online supplement (Data S1) alongside a more detailed discussion of the clinical and health‐care implications of this algorithm.

## Author Contributions

All authors discussed and planned the content. A.P.H. drafted the manuscript, with initial input from S.J.F. All authors then reviewed and amended this draft, and all approved the final version.

## Funding

The authors have nothing to report.

## Conflicts of Interest

The authors declare no conflicts of interest.

## Supporting information


**Data S1:** Discussion (online supplement).

## Data Availability

Data sharing not applicable to this article as no datasets were generated or analyzed during the current study.
