# Supplementary material for: Algorithms in Allergy: Diagnosis of Concomitant Inducible Laryngeal Obstruction in Adults With Asthma
Source: Allergy. 2026 Jun 19;81(8):3017–20. doi: 10.1111/all.70425 (PMC13466250; doi:10.1111/all.70425)
Supplement: Supplementary file 1 [file ALL-81-3017-s001.docx]

**Algorithms in *Allergy*: diagnosis of concomitant inducible laryngeal obstruction in adults with asthma**

Andrew P Hearn, Claire Slinger, Jennifer M Butler, Jemma Haines, James Hull, Stephen J Fowler

**Discussion (online supplement)**

Patients with untreated ILO have high healthcare utilisation, including unnecessary corticosteroid exposure, inappropriate escalation to biologic therapy, repeated emergency department attendances and unnecessary escalation to ventilatory support or intubation.^1^ Repeated courses of oral corticosteroids (OCS) are associated with an increased risk of multiple adverse outcomes, with evidence suggesting that harm accrues after as few as four lifetime short courses.^2^ Early and accurate identification of ILO has the potential to mitigate these risks through targeted, non-pharmacological interventions.

The proposed algorithm emphasises optimisation of type 2 (T2) inflammatory suppression as the first step in working up the patient with potential co-existent ILO. T2 biomarkers are well established predictors of asthma exacerbation risk, symptom burden, and response to biologic therapies,^2,3^ and therefore suppression of T2 inflammatory pathways remains central to reducing asthma-related morbidity and mortality.

Alongside optimising conditions which may exacerbate ILO (gastro-oesophageal reflux, post-nasal drip, breathing pattern disorder) the mainstay of treatment is ILO-directed speech and language therapy, which is effective at reducing symptom burden healthcare utilisation.^4^ At present, access to provocation laryngoscopy is limited by the availability of appropriately trained clinicians and specialist services. Given the high prevalence of ILO among patients with asthma and its associated morbidity, expanding access to diagnostic laryngoscopy and integrated multidisciplinary pathways should be considered a priority for healthcare systems.

Future beneficial developments in this area would include the development of ILO-specific diagnostics, including those based on objective measures of symptoms (questionnaires), airflow and resistance (e.g. oscillometry), and responses to controlled inhalational challenges.

**Provocation laryngoscopy**

Provocation laryngoscopy is typically performed in a joint clinic by a speech and language therapist, and a respiratory physician.^5^ A flexible nasendoscope is advanced transnasally, providing an opportunity to assess the nasal passages for polyps, rhinitis and post-nasal drip, then positioned to obtain a full view of the larynx and supraglottic structures. Where indicated, SLTs can also assess swallowing mechanics during laryngoscopy, and advise appropriately in patients who describe altered swallowing sensations.

Glottic and supraglottic movements and laryngeal tension are observed at rest during tidal breathing. The patient then undergoes a series of voicing exercises whilst the larynx remains under direct visualisation (mechanical provocation). After each exercise the patient should be asked to take a breath in, at which point ILO may be observed.

If ILO is not observed, scent provocation follows. Where possible the patient brings a known personal trigger, otherwise a strong-smelling deodorant is sprayed with the aim of triggering the patient's typical breathlessness. Inspiratory glottic closure of ≥50% is considered diagnostic of ILO, and reproduction of symptoms without observed laryngeal narrowing excludes it. A pragmatic diagnosis can also be made where partial glottic closure is observed without full symptom replication, or sometimes where the provocation does not trigger symptoms at all, provided the clinical history is otherwise fully supportive. The effectiveness of rescue-breathing and laryngeal control techniques can then be assessed under direct visualisation, also facilitating biofeedback, supporting the patient’s understanding of the diagnosis and breath-control exercises.

**Speech and language therapy for inducible laryngeal obstruction**

Speech and language therapy (SLT) is the cornerstone of treatment for ILO and has been shown to reduce symptom burden and healthcare utilisation.^4^ Treatment is individualised; many patients require only a small number of sessions following diagnostic laryngoscopy, while others benefit from a more prolonged course. The approach should be holistic, addressing symptom management within the context of the patient's daily life. Helping patients differentiate ILO symptoms from asthma and other co-morbid conditions is a fundamental part of the work, as well as treating any accompanying dysphonia where indicated, particularly when talking may trigger ILO.

Core components include training in laryngeal release techniques and “rescue breathing” exercises to abort or attenuate symptomatic episodes.^6^ Sessions also address the treatable traits commonly contributing to laryngeal hyperresponsiveness (laryngopharyngeal reflux, gastro-oesophageal reflux, post-nasal drip, hydration, and habitual mouth breathing) alongside laryngeal hygiene advice and self-management strategies that allow patients to maintain progress between sessions. Systematic desensitisation using the patient’s usual triggers can also be instrumental in improving daily functioning and quality of life. In patients with co-existent breathing pattern disorder, joint sessions with respiratory physiotherapy can be particularly valuable, allowing breathing-pattern retraining and laryngeal control work to be integrated within a single appointment. In patients where anxiety and trauma are important factors, input from clinical psychologists, including joint work with SLT is important. In more treatment-resistant patients, therapeutic laryngoscopy with biofeedback can consolidate adherence to and proficiency with prescribed breathing techniques. A systematic review of non-pharmacological interventions for ILO supports this approach, with consistent improvements in symptom control and quality of life across the available studies, although the underlying evidence base remains limited.^4^

**References**

1	Haines, J. *et al.* Clinical Characteristics and Impact of Inducible Laryngeal Obstruction in the UK National Registry. *The Journal of Allergy and Clinical Immunology: In Practice* **12**, 1337-1343 (2024). <https://doi.org/10.1016/j.jaip.2024.01.030>

2	Price, D. B. *et al.* Adverse outcomes from initiation of systemic corticosteroids for asthma: long-term observational study. *J Asthma Allergy* **11**, 193-204 (2018). <https://doi.org/10.2147/jaa.S176026>

3	Meulmeester, F. L. *et al.* Inflammatory and clinical risk factors for asthma attacks (ORACLE2): a patient-level meta-analysis of control groups of 22 randomised trials. *The Lancet Respiratory Medicine* **13**, 505-516 (2025). <https://doi.org/10.1016/S2213-2600(25)00037-2>

4	Haines, J. *et al.* Systematic review of the effectiveness of non-pharmacological interventions used to treat adults with inducible laryngeal obstruction. *BMJ Open Respiratory Research* **9**, e001199 (2022). <https://doi.org/10.1136/bmjresp-2022-001199>

5	Haines, J. E., Karen; Murphy, Jen; Pargeter, Nicola; Selby, Julia; Slinger, Clare; Wilson, Heidi. The role of speech and language therapy in upper airway disorders within adult respiratory services. . (London, 2021).

6	Haines, J., Smith, J. A., Fowler, S. J. & Yorke, J. Upper Airway Control Therapy (U-ACT): The Development of a Non-Pharmacological Intervention for Inducible Laryngeal Obstruction. *Respirology* **30**, 935-948 (2025). <https://doi.org/10.1111/resp.70073>
